# Intravital Multiphoton Microscopy as a Tool for Studying Renal Physiology, Pathophysiology and Therapeutics

**DOI:** 10.3389/fphys.2022.827280

**Published:** 2022-03-24

**Authors:** Bruce A. Molitoris, Ruben M. Sandoval, Mark C. Wagner

**Affiliations:** Indiana Center for Biological Microscopy, Indiana University School of Medicine, Indianapolis, IN, United States

**Keywords:** proximal tubule, glomerular filtration, endocytosis, renal hemodynamics, fluorescent biomarkers

## Abstract

Intravital multiphoton microscopy has empowered investigators to study dynamic cell and subcellular processes *in vivo* within normal and disease organs. Advances in hardware, software, optics, transgenics and fluorescent probe design and development have enabled new quantitative approaches to create a disruptive technology pioneering advances in understanding of normal biology, disease pathophysiology and therapies. Offering superior spatial and temporal resolution with high sensitivity, investigators can follow multiple processes simultaneously and observe complex interactions between different cell types, intracellular organelles, proteins and track molecules for cellular uptake, intracellular trafficking, and metabolism in a cell specific fashion. The technique has been utilized in the kidney to quantify multiple dynamic processes including capillary flow, permeability, glomerular function, proximal tubule processes and determine the effects of diseases and therapeutic mechanisms. Limitations include the depth of tissue penetration with loss of sensitivity and resolution due to scattered emitted light. Tissue clearing technology has virtually eliminated penetration issues for fixed tissue studies. Use of multiphoton microscopy in preclinical animal models offers distinct advantages resulting in new insights into physiologic processes and the pathophysiology and treatment of diseases.

## Introduction

Intravital multiphoton microscopy (MPM) of the kidney has been conducted for 20 years (Dunn et al., 2002, 2021). During this time advances in optics, lasers, computer software and hardware have led to more powerful systems having improvements in sensitivity and speed leading to a wide variety of new techniques exploring questions *in vivo* that were before unapproachable. Intravital multi-photon microscopy allows for visualization and quantification of dynamic cellular processes in normal functioning and diseased cells *in vivo*. A wealth of fluorescent biomarkers utilizing, blue, green, red, and far-red emitting fluorophores now allow four channels to be viewed simultaneously in three dimensions (3D) over time resulting in four-dimensional data. This has markedly increased the ability to observe and relate events involving multiple cell types and or intracellular organelles. Several laboratories have pioneered approaches and taken advantage of the many of these technological advances to study kidney physiology and pathophysiology (Dunn et al., 2002). Our laboratory has been aided along the way by numerous scientific collaborations and a NIH supported O’Brien Center for the past 20 years (Dunn et al., 2021). Table 1 lists some of the processes that can be quantified. In particular, the ability to study, within the same nephron, the interdependent roles of the glomerulus and proximal tubule (PT) simultaneously has been an exciting development for our laboratory. This mini-review will highlight a number of the advantages, techniques developed and utilized to quantify various aspects of renal physiology, pathophysiology and drug therapies, and will end indicating some of the existing limitations and challenges to the field.

**TABLE 1:** Potential uses of multiphoton microscopes in kidney processes.

Dynamic cell specific events:	References
**Cellular labeling and uptake**	
Cell type specific:	
Epithelial	Tanner et al., 2005; Ashworth et al., 2007; Sandoval and Molitoris, 2017; Dunn et al., 2021
Endothelial	Dunn et al., 2002; Sutton et al., 2003; Molitoris and Sandoval, 2011; Desposito et al., 2021; Gyarmati et al., 2021a
Glomerular labeling	Hackl et al., 2013; Schiessl et al., 2020; Gyarmati et al., 2021a
Uptake site:	
Apical	Dunn et al., 2002, 2021; Sandoval et al., 2004, 2012; Sandoval and Molitoris, 2017
Basolateral	Horbelt et al., 2007
**Mechanism:**	
Endocytosis	Dunn et al., 2002; Sandoval et al., 2004, 2019; Kalakeche et al., 2011; Wagner et al., 2016a; Sandoval and Molitoris, 2017; Schuh et al., 2018
Carrier/transporter mediated	Horbelt et al., 2007
Cell number	Hackl et al., 2013; Schiessl et al., 2020; Gyarmati et al., 2021b
Pattern distribution	Hackl et al., 2013; Schiessl et al., 2020; Shroff et al., 2021
**Cellular distribution**	
Site specific intracellular organelle accumulation	Weinberg and Molitoris, 2009; Hall et al., 2013
Cytosol accumulation	Molitoris and Sandoval, 2006
**Cell function**	
Endocytosis-quantitative analysis	Sandoval and Molitoris, 2008, 2017; Schuh et al., 2018; Sandoval et al., 2019
Intracellular trafficking	Sandoval et al., 2004, 2012; Molitoris and Sandoval, 2006; Sandoval and Molitoris, 2017
Transcytosis/exocytosis	Sandoval et al., 2012
Renin secretion	Schiessl et al., 2020
**Dynamic structural/functional effects within the kidney:**	
**Glomerular:**	
Size/volume	Sandoval et al., 2019
Permeability	Russo et al., 2007b; Nakano et al., 2012; Sandoval et al., 2012, 2014, 2019; Sandoval and Molitoris, 2013, 2014, 2017; Schiessl and Castrop, 2013; Dickson et al., 2014; Schiessl et al., 2015; Wagner et al., 2016a,b; Kidokoro et al., 2019; Gyarmati et al., 2021a
Fibrosis/Sclerosis	Ranjit et al., 2016
snGFR	Kang et al., 2006; Kidokoro et al., 2019
Afferent arteriole	Kang et al., 2006; Hackl et al., 2013; Gyarmati et al., 2021b
Macula densa	Shroff et al., 2021
**Microvasculature:**	
Blood flow rate	Molitoris and Sandoval, 2005; Sharfuddin et al., 2009; Sandoval and Molitoris, 2017; Sandoval et al., 2019
Endothelial permeability	Molitoris and Sandoval, 2011; Sandoval and Molitoris, 2017; Sandoval et al., 2019
WBC adherence/rolling/tissue invasion	Sandoval and Molitoris, 2017; Sandoval et al., 2019
Vasoconstriction	Kidokoro et al., 2019
**Epithelial cell:**	
Cell injury in necrosis, apoptosis	Dunn et al., 2002; Kelly et al., 2003; Ashworth et al., 2007; Kalakeche et al., 2011; Sandoval and Molitoris, 2017
Surface membrane/blebbing	Tanner et al., 2005; Ashworth et al., 2007
Tubular flow	Ferrell et al., 2015

**FIGURE 1:**
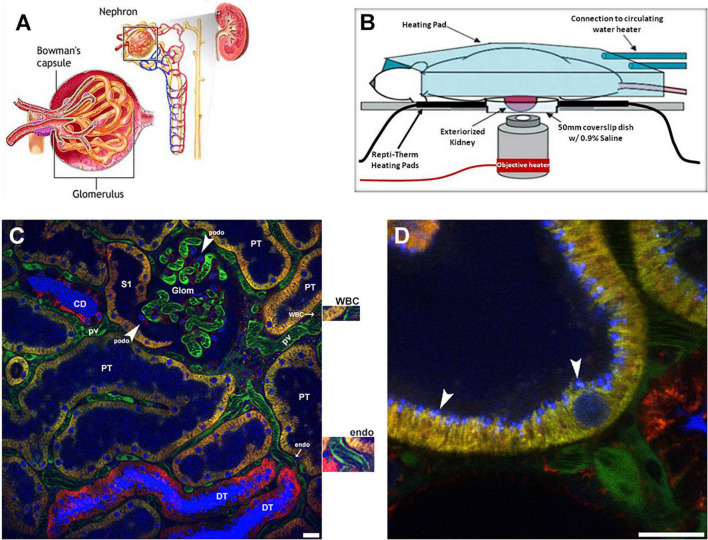
Visual resolving power of intravital multi-photon microscopy: a schematic of the renal architecture to subcellular resolution in proximal tubules. **(A)** Shows a classic diagram of the kidney in cross-section (upper right), with an inset of the complete nephron (center), and glomerulus (left). **(B)** Shows a schematic of the set-up used for 2-photon imaging. Placing the left exteriorized kidney onto a coverslip bottom dish on an inverted microscope is the most efficient way to minimize motion artifact from breathing. The various heating elements shown are used to maintain the proper kidney and body temperature, which is monitored and regulated. **(C)** Shows a single plane cross section with a glomerulus (glom) in the upper center and surrounding peritubular vasculature made visible using a large 150 kDa FITC dextran (green) retained in the plasma. Faint blue fluorescence in proximal tubule lumens (PT) and intense blue fluorescence in the lumen of collecting ducts (CD) and distal tubules (DT) comes from a small rapidly filtered 10 kDa Cascade Blue dextran bolus administered earlier. In DT and CD segments water removal concentrates the dextran intensifying the color. Three different mitochondrial dyes are seen in this image. Rhodamine 123 (yellow) predominantly labels the mitochondria of proximal tubule cells (PT); the open S1 PT segment is seen juxtapose to the glomerulus on the left. Tetramethylrhodamine methyl ester (TMRM, red) predominantly labels mitochondria in the distal tubules (DT) and collecting ducts (CD). Note the heterogenous labeling seen in the collecting ducts between the intercalated and principal cells. Finally, rhodamine B hexyl ester (also red) labels circulating white blood cells (WBC), podocytes (podo) in the glomerulus, endothelial cells (endo) see surrounding the peritubular vasculature, and a variety of cells in the interstitial space. Enlargements for the white blood cell and endothelial cell staining with rhodamine B hexyl ester are immediately adjacent to the right of the image. Hoechst 33342 labels the nuclei of all cell types (blue-cyan) in this micrograph (Bar = 20 μm). **(D)** Shows a high magnification micrograph of a shallow 5 μm, 3D reconstruction of proximal tubule from the same series in **(C)**. Endocytosis of the 10 kDa Cascade Blue dextran by the proximal tubule is seen accumulating at the sub-apical space in discrete blue vesicles (arrowheads). Note the various sizes of endosomes the microscope can resolve (Bar = 20 μm).

We will start by giving an overview of the imaging set up used, and some basics of what can be visualized and then proceed to individual structural components of the nephron (Figure 1A). The set up developed and utilized by our laboratory is shown in Figure 1B. We prefer the inverted microscope as experience has taught us that we can limit motion more thoroughly using this approach. Maintaining body temperature, volume status and appropriate anesthesia are essential to a successful study. We usually have an IV infusion ongoing and also measure the arterial blood pressure with a transducer to insure adequate hydration and physiologic parameters. Maintaining adequate anesthesia, but not too much, minimizes movement which is essential. We prefer inhaled anesthetics as they allow for fine tuning of the state of anesthesia. Figure 1C shows a low power view of the outer cortex of a Munich Wistar Frömter rat revealing a surface glomerulus surrounded by numerous tubules. Tubule types such as proximal tubules are identified by their endogenous autofluorescence and apical brush border membrane. Collecting ducts and distal tubules are indistinguishable from each other as they lack endogenous autofluorescence or any other visible landmark and appear as large empty patches similar in size to proximal tubules. Surrounding the tubules is the interstitial space containing dendritic cells and other cell types, especially during and following injury, and a network of peritubular capillaries and erythrocytes and white blood cells appearing as dark objects as they do not take up the fluorescent molecule contained in the plasma. Large molecular weight fluorescent molecules, that remain stable in the vasculature, are used to demarcate vessels, evaluate permeability and localize the interstitial space (Figure 1C). Figure 1 also shows a high magnification micrograph of a shallow 5 μm, 3D reconstruction of proximal tubule from the same series in Figure 1C. A 10 kDa filtered blue dextran is seen in early endosomes in the sub-apical region. The techniques and probes to be described have allowed us to understand normal renal physiology, the effect and pathophysiology of disease processes and the mechanisms of effective therapies.

## Renal Blood Flow Dynamics

Intravital MPM reveals a heterogeneous landscape of normal red blood cell flow within the peritubular vasculature and glomerular capillary loops. It has allowed important insights into ischemic and septic injury to the microvasculature. Large molecular weight fluorescent molecules create shadows of blood cells in the vasculature and the velocity of these cells can be inferred from the angles of these streaks in 2D images, or more accurately from the angle of the streaks in kymographs derived from line scans. This allows for assessment of red blood cell (RBC) flow rates and the degree of white blood cell rolling and attachment following ischemic injury and during sepsis (Dunn et al., 2002, 2021; Molitoris and Sandoval, 2005, 2011; Sharfuddin et al., 2009; Sandoval and Molitoris, 2017; Sandoval et al., 2019). In disease states red blood cells can stack together to form rouleaux reducing their oxygen delivery capacity and resulting in partial or complete peritubular capillary obstruction. These structures are easily identified, often lodged behind adherent white blood cells in the microvasculature, and can exit the kidney in the venous outflow (Sharfuddin et al., 2009; Molitoris and Sandoval, 2011; Sharfuddin and Molitoris, 2011). They may lodge in other microvascular beds in distant organs but the importance of this has not been determined. This has been used extensively to visualize and quantify the changes in peritubular capillary blood flow rates and microvascular dropout following ischemic injury (Basile, 2019).

Labeling White Blood Cells (WBC) nuclei using Hoechst 33342, and using distinctive nuclear morphology, gives a qualitative idea of the number and types of WBC flowing freely within the renal vasculature or found within the interstitium. In disease or injury models activated WBCs adhere to or roll along endothelial cells reducing flow (Sharfuddin et al., 2009; Molitoris and Sandoval, 2011; Sharfuddin and Molitoris, 2011). This can be visualized in the peritubular capillaries of S1 and S2 PT segments. Unfortunately, due to limited depth penetration, the S3 segment of PT cannot be visualized using intravital MPM. The S3 PT nephron segment is known to suffer the greatest capillary injury and microvascular dropout in ischemic models (Sharfuddin and Molitoris, 2011; Basile, 2019).

Ratiometric imaging of two non-overlapping fluorescent vascular dyes has been used in pre-clinical studies to determine glomerular filtration rates in rats under physiologic and following ischemic acute kidney injury (Yu et al., 2005; Wang et al., 2010). The glomerular sieving coefficient (GSC) of a fluorescent compound is the ratio of fluorescence in Bowman’s Space divided by fluorescence in the glomerular capillary plasma. A small 5 kDa dextran, with a GSC of 1.0 is rapidly and completely filtered across glomerular capillaries, and a large dextran, 150 kDa, has a very low GSC and is retained and stable in the vasculature. This approach has been adapted to clinical studies to provide both quantitative GFR and plasma volume determinations (Rizk et al., 2018; Molitoris et al., 2019).

## Glomerular Imaging

In MWF rats surface glomeruli are easily identified allowing the dynamic aspects of glomerular capillary vessel diameter, RBC flow rates, single nephron GFR, and permeability to be quantified (Dunn et al., 2002, 2021; Sandoval et al., 2019). Of these different parameters the measurement of glomerular permeability of macromolecules is likely the most clinically important and has created controversy in the literature. Previous methods to quantify glomerular permeability were based on micropuncture or urinary fractional clearance studies. These techniques compare tubular lumen filtrate and urinary concentrations to plasma concentrations, respectively. Unfortunately, there is no allowance for PT mediated loss of material from the lumen due to tubular reabsorption via fluid phase or receptor mediated endocytosis prior to the collection location (Russo et al., 2007a,b, 2009; Sandoval et al., 2012; Sandoval and Molitoris, 2013; Wagner et al., 2016a). Since the early S1 segment is primarily responsible for albumin reabsorption, micropuncture studies often miss the most endocytic S1 portion thus underestimating the amount of filtered albumin due to the needle placement away from Bowman’s Space. Thus, our MPM studies have shown the glomerular sieving coefficients (GSCa) higher than most micropuncture studies, in the 0.012–0.015 range (Sandoval et al., 2012; Sandoval and Molitoris, 2013), while micropuncture studies have for the most part found values in the 0.0005 range for MWF rats. A recent micropuncture study did show a much higher GSCa (Hu et al., 2016). Russo et al. (2007b, 2009) used Munich Wistar Simonsen rats and found their GSCa was in the range of 0.025–0.030. The reason for the GSCa differences in MWF and MWS has not been investigated, but we have validated the high GSCa in MWS rats. Interestingly, early streptozocin diabetic Munich Wistar Simonsen rats had no change in their GSCa, but showed reduced PT reabsorption of filtered albumin and increased urinary albumin (Russo et al., 2009).

Unfortunately, glomerular studies in mice are challenging as glomeruli are rarely within 100 microns of the surface after 4 weeks of age in all mice strains (Schiessl et al., 2013). To circumvent this challenge prolonged ureteral obstruction, or the use of non-steroidal anti-inflammatory agents, have been used to force glomeruli to the surface secondary to cortical destruction. However, this approach is known to cause excessive inflammation, fibrosis, and loss of proximal tubule (PT) structure and function leaving interpretation of the results problematic especially when studying a disease model (Chevalier et al., 2009; Yang et al., 2010).

To get accurate GSCa using MPM one must have the necessary sensitivity to correct for measurement and subtraction of background values (Yang et al., 2010; Sandoval et al., 2014; Sandoval and Molitoris, 2014). Setting the background too high lessens sensitivity and reduces the GSCa. This requires using the full dynamic range of the system’s detectors and particularly the correct offset or black level. Setting the black level for Bowman’s Space to zero to eliminate all background signal, markedly reduces detector sensitivity and distorts the results of the low intensity signals (Sandoval et al., 2014; Sandoval and Molitoris, 2014). The old adage, your results are only as good as your sensitivity holds in this situation.

## Proximal Tubule Endocytosis and Transcytosis

Proximal tubules function to reabsorb filtered fluid, electrolytes and macromolecules to prevent loss via urinary excretion. They also function to “sense” the internal environment and have immunologic surveillance capabilities (Hato et al., 2013). Intravital MPM has played an important role in understanding the processes involved, intracellular trafficking and catabolism of the reabsorbed material (Molitoris and Sandoval, 2005; Horbelt et al., 2007; Sandoval and Molitoris, 2008, 2017; Sandoval et al., 2019). This has been particularly important for macromolecules including therapeutic agents (Sandoval et al., 2004; Molitoris and Sandoval, 2006; Molitoris et al., 2009; Kalakeche et al., 2011). Kinetic studies with p53 fluorescent siRNA showed PT endocytosis, cytosolic delivery and a short intracellular half-life corresponding to the rate and duration of the synthesis of p53 (Molitoris et al., 2009).

Macromolecule reabsorption across the apical membrane occurs via receptor mediated and fluid phase endocytosis, Figure 1D. The Hall laboratory, using a tissue clearing technique to allow for deeper MPM penetration in fixed tissue showed the S1 segment uses receptor mediated endocytosis (RME) primarily whereas the S2 and S3 segments primarily use the fluid phase endocytosis (FPE; Schuh et al., 2018). Thereafter endocytic trafficking sorts material into two main pathways, lysosomal for catabolism and transcytosis for reclamation. Intravital MPM has helped expand the investigative focus, beyond glomerular dysfunction, to elucidate the role tubular injury plays in proteinuric and albuminuric diseases previously thought to be associated solely with damage to the filtration barrier (Sandoval et al., 2012; Sandoval and Molitoris, 2013; Wagner et al., 2016a). In quantifying uptake it is important not to saturate the intensity of the endosomal pool (particularly lysosomes) as this will underestimate the amount of the material therein (Sandoval and Molitoris, 2008, 2014; Sandoval et al., 2014).

Careful consideration to the background fluorescence must also be accounted for when quantifying uptake of any compound into the lysosomal/endosomal pool. This value must be subtracted from the raw images to determine true and meaningful intensity values (Sandoval et al., 2019). We typically take three background 3D volumes of proximal tubules at different laser transmissivities prior to imaging, and calculate average intensity values at each laser power to mathematically compensate for saturating intensities. This generates intensity correction factors between the different laser powers used to normalize background subtracted images taken at different laser powers. Thresholding is used to help correct for autofluorescence in lysosomes, and is fluorophore channel specific (Sandoval and Molitoris, 2013; Sandoval et al., 2019).

Transcytosis has not been extensively studied in PT cells *in vivo* because it is difficult to characterize the amount of transcytosis based on basolateral transport into the interstitial space (Sandoval et al., 2012; Dickson et al., 2014). We observed albumin transcytosis via both finger like projections and vesicles from endosomal accumulations reaching basolateral membranes of proximal tubules (Sandoval et al., 2012). This is in agreement with FcRn mediated immunoglobulin transcytosis in cultured cells (Ward et al., 2005). Transcytosis of albumin was confirmed using molecular techniques, but the amount of albumin undergoing transcytosis remains unknown (Tenten et al., 2013). A potential role for PT sorting of glycated, carbamylated and other potentially toxic albumins, mediated by FcRn binding, for catabolism via lysosomal trafficking has been proposed as a mechanism to rid the body of altered albumins while preserving physiologic albumin for transcytosis (Dickson et al., 2014; Wagner et al., 2016b; Yadav et al., 2021). Proximal tubule transcytosis of folic acid and other vitamins is known to occur (Sandoval et al., 2004). Transcytosis from the basolateral membrane has also been demonstrated for PT cells using other techniques (Hu et al., 2016).

## Mitochondrial Structure and Function and Associated Processes

Mitochondrial structure and function can both be studied using intravital MPM in multiple cell types simultaneously. Multiple cell membrane permeable dyes can be used to determine the mitochondrial potential and follow its loss during injury (Weinberg and Molitoris, 2009; Hall et al., 2013). These studies identified the relative resistance in cellular mitochondrial potential to ischemia among the different tubular epithelial cells and structural changes in PT mitochondria. Three different mitochondrial dyes are used to label various cortical cells (Figure 1C). Rhodamine 123 predominantly labels proximal tubule cells, Tetramethylrhodamine methyl ester (TMRM), labels the collecting ducts and distal tubule cells. However, increased loading concentrations can cause accumulation in other tubule types. A second red dye, Rhodamine B hexyl ester, is used to stain mitochondria of endothelial cells, podocytes, circulating white blood cells, and cells within the interstitial space. All three dyes can be used simultaneously at lower loading concentrations, even the two red dyes because of the disparate cell types they label (Hall et al., 2013). The differences in cells labeling by each dye may relate to the organic ion transport processes in each cell type.

Apoptosis is another intracellular process that can easily be followed and quantified using MPM and can be differentiated from necrosis using Hoechst 33342 and the vital dye propidium iodide (Dunn et al., 2002, 2021; Kelly et al., 2003). Bright condensed staining along the edge of the nucleus, as well as bright fragmented structures, are hallmark changes that occur during apoptosis. Staining of nuclei with propidium iodide is indicative of a necrotic cell with a compromised cell membrane as this dye is membrane impermeant.

## Alternative Methods of Probe Delivery

While intravascular delivery remains the mainstay for delivering fluorescent biomarkers to the kidney, a major advantage of MPM is the ability to pair other techniques with it and observe the subsequent process *in vivo* using a biomarker that cannot be delivered via the vascular route. We have used micropuncture techniques to deliver plasmids to fluorescently label cellular actin structures (Tanner et al., 2005; Ashworth et al., 2007), deliver fluorescently labeled bacteria to the lumen of PT cells to follow growth, invasion and cellular responses of PT (Mansson et al., 2007; Melican et al., 2011; Choong et al., 2012; Schulz et al., 2018), to endothelium and WBC (Sutton et al., 2003; Molitoris and Sandoval, 2011) and hydrodynamic delivery of genes to cells throughout the kidney (Kolb et al., 2018). These techniques are done just prior to imaging the animal or on the microscope stage during imaging. The ability to follow the result in a small area of cells eliminates the need to deliver the probe to the entire kidney.

## Challenges to Studying the Kidney

**FIGURE 2:**
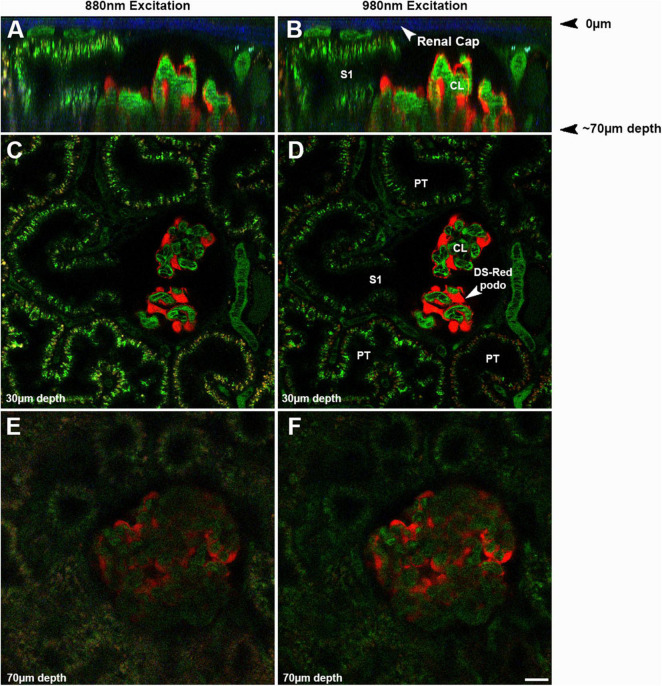
Effect of imaging depth on image intensity and resolution: The same glomerulus in a strain of Munich Wistar Frömter rats, expressing the fluorescent protein DS Red selectively in podocytes, was imaged from 20 to 70 microns from the surface using two different wavelengths, 880 **(A,C,E)** and 980 nm **(B,D,F)**. Oregon Green 488 labeled rat serum albumin (OG488-RSA) was injected I.V. and can be seen in the vasculature, proximal tubules, and glomerulus. To assure illumination remained relatively constant from the upper to the lower optical sections in the image volume, the Linear Z-Compensation feature on the Leica Dive Multi-Photon system was utilized. **(A,B)** Show orthogonal the Linear Z-Compensation feature on the Leica Dive Multi-Photon system X-Z projections of a 73 μm micron volume, with the glomerular surface at the top of the image; note the 2nd harmonic excitation of collagen in the renal capsule (arrows, blue). These orthogonal projections in show the degradation in resolution and intensity in the deeper portions of the tissue, due to light scattering of the emitted light. The S1 cross section from both excitation wavelengths can resolve small endosomes and tubular structures rich in OG488-RSA at the upper cross section at 30 μm. The lower cross section of the same S1 has a hazier appearance, with only a few individual endosomes identifiable. Single plane images shown in **(C–F)** show the loss in resolution and intensity of the individual endosomes in **(E,F)** (taken at 70 μm), as compared to **(C,D)** (taken at 30 μm). The loss in resolution at the lower depths extends to small structures like endosomes and also includes losing the ability to discern circulating red blood cells in peritubular vessels and glomerular capillary loops. The inability to clearly discern the boundaries of glomerular capillary loops or peritubular blood vessels and the general haze makes intensity based (such as GSCs) or morphology based (such as RBC flow) analysis nearly impossible and greatly increases error and data variability (Bar = 20 μm).

Imaging the kidney intravitally has a number of challenges that must be understood and minimized. The kidney has reduced optical penetration, compared to the many tissues, due to increased blood flow, cellular heterogeneity, and inherent autofluorescence. This results in scattering and absorption of the emitted light. This limits the depth of penetration allowing for high sensitivity and resolution to less than 100 microns whereas studies in brain can penetrate over a millimeter (Sandoval et al., 2012; Sandoval and Molitoris, 2013). Figure 2 shows the effect of imaging depth on sensitivity and resolution from 20 to 70 μm utilizing two different wavelengths, 880 and 890 nm, even when using the Linear Z-Compensation feature on the Leica Dive Multi-Photon system. Orthogonal views, Figures 2A,B and single plane images Figures 2C–F show the drop off in both sensitivity and resolution regardless of the wavelength used. As we have shown before, this drop off is greater for fluorophores emitting in the green spectrum due to enhanced quenching by hemoglobin (Sandoval et al., 2012; Sandoval and Molitoris, 2013).

Two approaches have recently been advanced to allow for deeper penetration and visualization. Schuh et al. (2016) using specialized longer wavelength excitation lasers and far-red probes, demonstrated greater depth advantages when conducting intravital 2 and 3-photon studies of the kidney. Adaptive optics may also be able to improve the depth of penetration by compensating for system and sample aberrations in the excitation beam wavefront. This will improve the focus resulting in higher intensities and improved spatial confinement at depth (Ji, 2017). However, adaptive optics has not been applied to imaging the kidney.

It is also more difficult to stabilize the kidney leading to increased motion artifacts. These challenges and approaches to minimize them have been carefully described previously (Dunn et al., 2002, 2021; Sandoval and Molitoris, 2017; Sandoval et al., 2019).

The use of fluorescent probes or biomarkers to delineate aspects of glomerular filtration, peritubular capillary function and tubular function in health and disease is critical but not without challenges. For instance, commercially available fluorescent dextrans all too often have a wide molecular weight dispersion limiting their accurate characterization of processes such as glomerular permeability (Sandoval et al., 2012; Sandoval and Molitoris, 2013). We have solved this problem by first obtaining a highly uniform dextran, with low MW dispersion, and second by performing the fluorophore conjugation directly (Sandoval et al., 2012; Sandoval and Molitoris, 2013, 2017).

Measurement of the fluorescence intensity of labeled compounds is the basis for many quantitative studies including glomerular permeability, PT reabsorption, co-localization and many others. Quantitative intensity-based data analysis requires strict attention to how instrument parameters and sensitivity are managed to completely utilize the full dynamic range of the system (Sandoval and Molitoris, 2013; Sandoval et al., 2014, 2019). For example, if settings are not correct the ratiometric intensities of the same compound in two different compartments can vary by orders of magnitude. Full dynamic range utilization requires system detectors with correct offset, or black level settings, showing only a few pixels in the image randomly flash as having values of zero (Sandoval et al., 2014; Sandoval and Molitoris, 2014). When acquiring background images setting all pixels to zero, in an effort to remove background during acquisition, decreases sensitivity thus reducing the ability to detect low intensity values (Nakano et al., 2012; Schiessl and Castrop, 2013; Sandoval and Molitoris, 2014; Schiessl et al., 2015).

When studying a protein it is essential to make sure the conjugation of the fluorophore does not alter its physiologic binding and or function. For albumin we have found a 1:1 ratio of protein to fluorophore and use of a multi-carbon spacer on the fluorophore, maintains physiologic binding affinity (Wagner et al., 2016a). Increasing the conjugation ratio often leads to reduced function and altered kidney metabolism and vascular clearance (Wagner et al., 2016a). Therefore, it is essential, but often overlooked, to ask, and test if possible, whether the labeled protein has the same biological properties as the native protein before undertaking imaging studies (Wagner et al., 2016a; Sandoval et al., 2019).

The answer to which animal model to use is primarily dictated by the question being asked. Mice have several advantages including a wealth of transgenic strains and many strains with fluorescently labeled cells such as the Tie-2 mice labeled endothelial cells. The relative ease of generating unique transgenic mice has also been an important advantage. The Peti-Peterdi laboratory has used this approach to follow endothelial and glomerular epithelial regeneration using serial intravital multi-photon microscopy (Hackl et al., 2013; Schiessl et al., 2020; Desposito et al., 2021). These studies have shed light on the dynamic alterations, spatial distribution and fate of single renal cells or cell populations and their migration patterns in the same tissue region over several days in response to various stimuli. As delineated above, glomerular studies in mice are challenging as glomeruli are rarely within 100 microns of the surface after 4 weeks of age in all mice strains (Schiessl et al., 2013). Ureteral obstruction for 6–12 weeks, or non-steroidal anti-inflammatory agents, have been used to induce surface glomeruli, but this comes at the cost of tubular destruction, cortical atrophy and fibrosis (Chevalier et al., 2009; Yang et al., 2010). This same team of investigators have shown, in their recent studies, deep glomeruli in mice can be imaged and both afferent and efferent arteriolar RBC flow can be quantified (Gyarmati et al., 2021a,b; Shroff et al., 2021). To do this they again used longer wavelength light to visualize normal cortical depth glomeruli in mice. However, resolution does suffer and not all processes can be quantified at this depth. A recent study also shows mice glomeruli may increase on the surface during progressive disease in a mouse model of Alport’s Syndrome (Gyarmati et al., 2021b).

Another investigative team has used cortical resection to expose subsurface glomeruli in mice. While necessary for glomerular visualization, this approach resulted in a very high GSC for albumin, the ratio of glomerular filtrate to capillary albumin fluorescence, of 0.2–0.3, likely resulting form injury induced by the resection (Kidokoro et al., 2019). We have chosen to primarily study Munich Wistar Frömter rat strains (Simonsen and Frömter) that have easily imaged surface glomeruli. The Peti-Peterdi laboratory has developed a nice technique to quantify single nephron GFR and renal blood flow in these rats (Kang et al., 2006). The Frömter strain has up to three times more than the Simonsen’s strain. Surface glomerular capillaries are seen within Bowman’s Capsule, lack any autofluorescence, and are surrounded by proximal tubules (Figures 2C,D). The rat glomerulus consists of lobules and is about 100 microns in diameter allowing full 3D studies to be conducted. Unfortunately, the afferent arteriole usually lies at the bottom of the glomerulus making studies of it difficult due to decreased resolution and sensitivity at that cortical depth.

The S1 segment of the proximal tubule can be easily identified having a direct opening into the glomerulus making identifying and studying this unique and very endocytic segment easy (Dunn et al., 2002; Molitoris and Sandoval, 2005, 2011; Yu et al., 2005; Russo et al., 2007a; Sharfuddin et al., 2009; Wang et al., 2010; Sharfuddin and Molitoris, 2011; Sandoval and Molitoris, 2013, 2017; Dickson et al., 2014; Rizk et al., 2018; Basile, 2019; Molitoris et al., 2019; Sandoval et al., 2019). Being able to identify and study the S1 segment of the proximal tubule is important as this segment has the greatest capacity for endocytosis of macromolecules. This includes filtered proteins, vitamins, drugs, and endogenous and exogenous nephrotoxins. Differentiating S1 from S2 PT can be done in mice based on endogenous autofluorescence but not in rats (Kalakeche et al., 2011). We have found that anionic and neutral dextrans are endocytosed differently between S1 and S2 thus providing another way to distinguish these PTs in rats.

## Conclusion

In summary, intravital MPM can serve as an invaluable tool to enhance the research objectives of many laboratories studying the physiology, pathophysiology and therapy of the kidney, or any organ that is accessible to exposure, placement and stabilization for intravital MPM microscopy. Multiple aspects can be studied individually and up to four fluorescent dyes can be visualized and spectrally separated. Since these dyes localize differently within tissue compartments, a greater number of cellular of processes can be simultaneously studied than the number of detector channels.

## Author Contributions

BM designed the study. BM, RS, and MW wrote the manuscript. RS created the figure. All authors contributed to the article and approved the submitted version.

## Conflict of Interest

The authors declare that the research was conducted in the absence of any commercial or financial relationships that could be construed as a potential conflict of interest.

## Publisher’s Note

All claims expressed in this article are solely those of the authors and do not necessarily represent those of their affiliated organizations, or those of the publisher, the editors and the reviewers. Any product that may be evaluated in this article, or claim that may be made by its manufacturer, is not guaranteed or endorsed by the publisher.
